# Transcriptomics Data Mining to Identify Novel Regulatory Genes of Iron Uptake in Drought-Stressed Wheat

**DOI:** 10.3390/ijms262210955

**Published:** 2025-11-12

**Authors:** Mohamed Najib Saidi, Omeima Rebai, Fadhila Hachani, Gianpiero Vigani, Stefania Astolfi

**Affiliations:** 1 Biotechnology and Plant Improvement Laboratory, Centre of Biotechnology of Sfax, P.O. Box 1177, Road Sidi Mansour 6 km, Sfax 3018, Tunisia; omeimarebai@yahoo.fr (O.R.); fadhylaaha@gmail.com (F.H.); 2 Department of Life Sciences and Systems Biology, University of Torino, 10124 Torino, Italy; gianpiero.vigani@unito.it; 3 Department of Agricultural and Forest Sciences (DAFNE), University of Tuscia, 01100 Viterbo, Italy

**Keywords:** drought, Fe deficiency, co-expression, transcription factor, durum wheat

## Abstract

Understanding the molecular crosstalk between drought and iron (Fe) homeostasis is crucial for developing drought-tolerant wheat cultivars with enhanced nutrient quality. In this study, transcriptomic data mining identified 23,271 and 5933 differentially expressed genes (DEGs) under drought and Fe deficiency, respectively, with 2479 DEGs in response to both stresses. Notably, this overlapping set included significant numbers of genes encoding transcription factors (TFs) (149 genes), Fe homeostasis components (274 genes), and those involved in phytohormones pathways (245 genes), particularly the abscisic acid (ABA) pathway. Gene Ontology (GO) analysis revealed specific and commonly affected biological processes, such as response to abiotic stimulus and heme binding. Furthermore, co-expression network analysis revealed modules highly enriched with genes involved in transcriptional regulation and Fe uptake, enabling the identification of key hub regulatory genes, belonging to the MYB, NAC, BHLH, and AP2/ERF families, involved in the shared stress response. Finaly, the expression of a set of candidate TF-encoding genes was validated using qRT-PCR in durum wheat under drought and Fe starvation, providing a detailed overview of the possible shared regulatory mechanisms linking drought and Fe deficiency responses.

## 1. Introduction

Wheat (*Triticum aestivum* L.) is one of the major cereal crops cultivated in the world and provides about 20% of daily calories and protein intake in the human diet, especially in the Mediterranean area [1]. Indeed, wheat is a valuable source of vitamin B, dietary fiber and essential minerals, including iron (Fe), magnesium, manganese, phosphorus, and zinc [2]. However, extreme climatic conditions, such as drought, significantly impact wheat grain composition and quality [3].

The growth and yield of wheat are highly susceptible to various biotic and abiotic stresses. Moreover, drought is considered as a major concern particularly in the Mediterranean regions where altered precipitation patterns due to climate change exacerbate its impact. Moreover, drought severely affected grain yield by impairing several physiological processes such as through a decrease in nutrient assimilation, embryogenesis, endosperm development, and seed growth [4]. Recent evidence revealed that drought tolerance in durum wheat is linked to mineral nutrient plasticity, suggesting that investigating nutrient variation is crucial to understand drought-induced responses in plants [5,6].

Among mineral nutrients, Fe is an essential micronutrient for plants and plays a pivotal role in several key metabolic processes, including photosynthesis, chloroplast formation, chlorophyll synthesis, electron transport, and redox reactions [7]. Beyond its metabolic functions, Fe is critical for mitigating various stresses, such as drought, salinity, and heavy metal toxicity [8]. Notably, Fe plays a vital role in activating antioxidant enzymes, such as catalase, which are essential for protecting plant cells from oxidative damage [8].

To cope with the limited solubility and poor availability of Fe in soils, plants have developed sophisticated adaptive strategies for regulating their uptake, storage, and metabolism. In fact, two main strategies (Strategies I and II) have evolved in plants for acquiring Fe from the rhizosphere [9]. Strategy I (reduction strategy) is prevalent in most plant species and has been well characterized in the model plant *Arabidopsis thaliana*. Strategy II, known as the chelation-based strategy, is restricted to grasses and involves organic molecules such as phytosiderophores (PSs) and the YS/YSL family transporters in roots [10].

Furthermore, the regulation of gene expression is a central process in plant adaptation to environmental stresses including drought and low Fe availability. Transcription factors (TFs) encoding genes are known as key regulators of plant response to low Fe availability by the induction of Fe acquisition genes. Indeed, several TF families, including MYB, bHLH, WRKY, and NAC, are known to regulate the response to Fe deficiency [11,12,13]. Several master regulators of Fe deficiency response have been identified across species in rice such as IDEF1, IDEF2, and IRO2 which positively regulate the Fe deficiency response, while *OsIRO3* acts as a negative regulator of several Fe-related genes [14]. Notably, *OsIDEF1* gene modulates the expression of *OsIRO2* and *OsIRO3* under Fe deficiency in rice [15]. In *Arabidopsis*, the MYB88 gene positively regulates the Fe deficiency response [16]. Consistently, the *myb8* knock-out mutant exhibited enhanced sensitivity to Fe deficiency, resulting in decreased root length, chlorophyll content, and Fe concentration [16]. Moreover, it was reported that the expression of several iron homeostasis genes (AHA2, FRO2, and IRT1) is regulated by bHLH TFs under Fe deficiency in *Arabidopsis* [17]. Conversely, two MYB TFs (MYB10 and MYB72) are induced by Fe deficiency and positively co-regulate the expression of two genes encoding for nicotianamine synthase NAS2 and NAS4 in rice [18].

Besides transcription factors, phytohormones are involved in the regulation of plant response to low iron availability. Indeed, auxin and ethylene positively regulate the Fe deficiency response [19] while cytokinin and jasmonic acid act as negative regulators [20,21]. Moreover, abscisic acid (ABA) and gibberellic acid are reported to enhance the Fe deficiency response in *Arabidopsis* [22]. Notably, ABA is a key phytohormone involved in drought response [23] by regulating several signaling and developmental processes, such as triggering stomatal closure to prevent transpirational water loss [24].

Although plants show distinct responses to Fe deficiency and drought, this latter significantly affects nutrient uptake by limiting nutrient diffusion between the soil and the root surface due to reduced soil moisture content [25]. Consequently, drought impairs overall plant nutrition, evidenced by decreased concentrations of nutrients like nitrogen and phosphorus [26]. Moreover, drought reduces nutrient uptake efficiency by reducing mineralization and altering the kinetics of root nutrient uptake [26]. This systemic overlap in nutritional impact strongly suggests a potential connection between these two stresses at the regulatory level.

Recently, transcriptomics data analysis mining has become a powerful tool to reveal plant resilience by elucidating the crosstalk between stress signaling pathways. Indeed, comparative RNA-seq data analysis generated from contrasting common bean genotypes successfully identified key regulatory genes related to drought tolerance [27]. Similarly, comparative transcriptome analysis of leaves and roots revealed key transcription factors, such as the bHLH family, regulating iron homeostasis under Fe deficiency in wheat [28]. However, our understanding of the molecular crosstalk between Fe deficiency and drought stress responses, particularly at the transcriptomic level in cereal crops, remains limited.

Our aim is to gain a comprehensive insight into the regulation of Fe uptake in wheat under limited-water conditions. Thus, we employed data mining of publicly available RNA-seq data to identify a set of specific and shared differentially expressed genes under drought and Fe deficiency in wheat. Moreover, Gene Ontology (GO) analysis revealed a crosstalk between transcription regulation, ion homeostasis, and phytohormones pathways. In addition, a gene co-expression network was constructed to reveal key hub transcription factors regulating Fe homeostasis under drought and/or iron deficiency. Finally, the expression of candidate TFs was validated in durum wheat subjected to drought and Fe deficiency using qRT-PCR. The candidate TFs genes linking Fe homeostasis and drought response serve as an important genetic resource for future investigations aiming to further decipher their roles using heterologous expression and for wheat breeding for simultaneously enhancing Fe uptake and drought tolerance in wheat.

## 2. Results

### 2.1. Identification of DEGs Under Drought and Fe Deficiency

**Figure 1:**
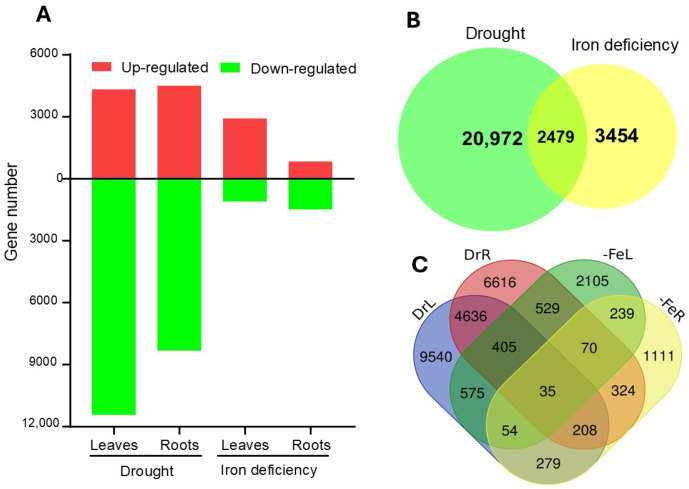
Global analysis of differentially expressed genes (DEGs). (**A**) Bar chart illustrating the total number of identified DEGs under drought stress and Fe deficiency. (**B**) Two-way Venn diagram showing the number of common DEGs between the two stress conditions. (**C**) Four-way Venn diagram detailing the tissue-specific overlap (leaves and roots) of DEGs under both drought and Fe deficiency.

Transcriptome data analysis using DEGseq2 identified a total of 23.271 and 5.933 DEGs (FC ≥ 2; FDR ≤ 0.05) under drought and Fe deficiency, respectively (Figure 1A; Supplementary Table S1).

Indeed, under drought stress, about 4000 genes were up-regulated in both leaves and roots while −11,435 and 8344 DEGs were down-regulated in leaves and roots, respectively (Figure 1). Considering iron deficiency, 2904 and 831 genes were up-regulated in leaves and roots, respectively. The leaves samples had less down-regulated genes (1108 genes) as compared to the roots, with 1488 down-regulated genes under Fe deficiency (Figure 1). Moreover, a two-way Venn diagram analysis showed that 2479 genes were differentially expressed under both stress conditions (Figure 1B), suggesting shared response between drought and Fe deficiency. On the other hand, four-way Venn diagram analysis revealed 35 genes that were differentially expressed in all tissues under both stress conditions (Figure 1C). Comparative analysis of DEGs across tissues showed 1069 and 637 genes commonly affected in leaves and roots, respectively. In leaves, 500 genes exhibited similar expression patterns under combined stress, with 268 up-regulated and 232 down-regulated genes. In roots, 49 genes were up-regulated, while 263 genes were down-regulated under drought and Fe deficiency (Supplementary Material Figure S1).

### 2.2. GO Enrichment Revealed Biological Process Related to Drought and Fe Uptake

**Figure 2:**
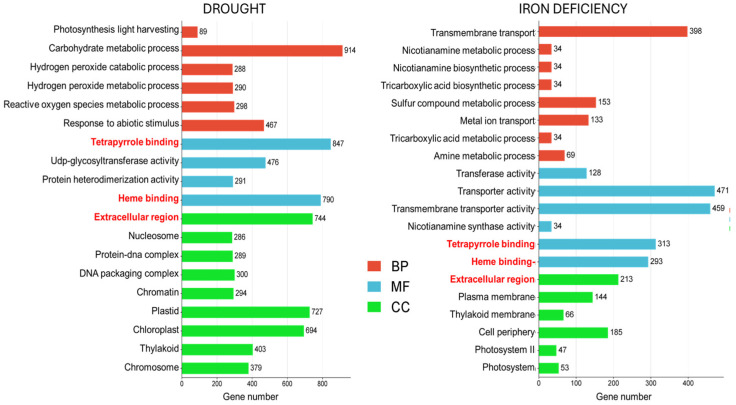
Gene Ontology (GO) enrichment analysis of DEGs. Enriched GO terms across three categories: biological processes (BP), molecular function (MF), and cellular component (CC), identified under drought and Fe deficiency stresses. The X-axis represents the number of genes with each term. Common GO terms are highlighted in red.

To gain deeper insight into the functional implications of the DEGs, GO enrichment analysis was performed. GO terms with a *p*-value < 0.05 were assigned to DEGs according to their biological process (BP), molecular function (MF), and cellular component (CC). Among the top 20 significantly enriched GO terms associated with drought stress, several categories were prevalent (Figure 2; Supplementary Material Table S2).

Among the most significant GO terms, processes related to photosynthesis such as “photosynthesis light harvesting” (GO:0009765) and general metabolism like “carbohydrate metabolic process” (GO:0005975) were identified. Furthermore, processes associated with abiotic stress response were significantly enriched, including “response to abiotic stimulus” (GO:0009628). Additionally, several GO terms related to oxidative stress such as ‘hydrogen peroxide catabolic process’ (GO:0042744), ‘hydrogen peroxide metabolic process’ (GO:0042743), and ‘reactive oxygen species metabolic process’ (GO:0072593) were enriched. Finally, the overrepresented GO terms within the MF category included ‘tetrapyrrole binding’ (GO:0046906), ‘UDP-glycosyltransferase activity’ (GO:0008194), ‘protein heterodimerization activity’ (GO:0046982), and ‘heme binding’ (GO:0020037) were also identified (Figure 2). Among the significantly enriched GO terms within the CC category, ‘extracellular region’ (GO:0005576), ‘nucleosome’ (GO:0000786), ‘protein-DNA complex’ (GO:0032993), and ‘DNA packaging complex’ (GO:0044815) were prominently represented.

Concerning Fe deficiency, the most overrepresented BP terms included ‘transmembrane transport’ (GO:0055085), ‘sulfur compound metabolic process’ (GO:0006790) and ‘metal ion transport’ (GO:0030001). Furthermore, significantly enriched MF terms under Fe deficiency included ‘transmembrane transporter activity’ (GO:0022857), ‘nicotianamine synthase activity’ (GO:0030410), and ‘heme binding’ (GO:0020037). The overall analysis of enriched GO terms under both drought and Fe deficiency revealed significant overlaps in several biological processes including photosynthesis, plant metabolism, response to abiotic stress, antioxidant system and heme binding. This finding suggested shared signaling pathways that were modulated by both drought and Fe deficiency.

### 2.3. Fe Homeostasis Genes Are Modulated Under Fe Deficiency and Drought Conditions

**Figure 3:**
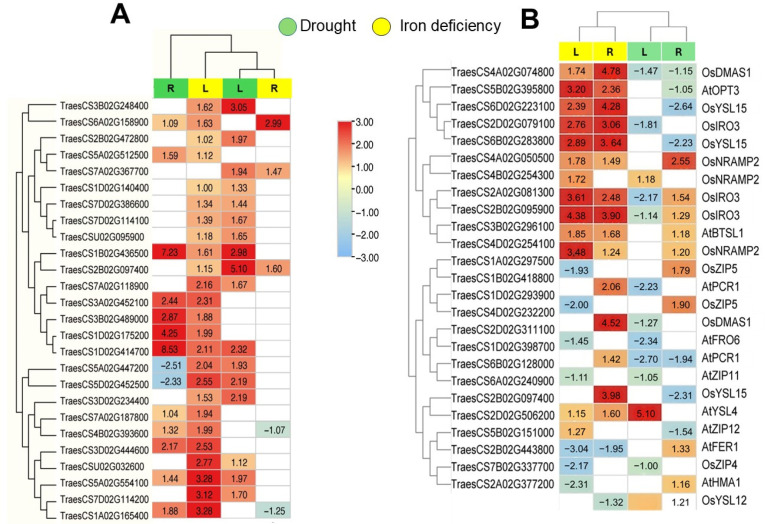
Expression profiles of ion transporters related genes. (**A**) Heatmaps showing the expression pattern (Log_2_FC) of most differentially expressed transporter encoding gene families shared among drought and Fe deficiency. (**B**) Expression pattern of Fe homeostasis genes and their orthologs from *Arabidopsis* and rice with known functions. Empty boxes indicate that the gene was not identified as DEGs in corresponding tissue.

Referring to genes annotation and conserved protein domain (InterPro IDs), 274 genes encoding for transmembrane channels and transporters were identified among the common DEGs. Indeed, 39 genes encoded for ABC transporters, 30 encode for Heavy-Metal-Associated (HMA) domain containing proteins, 25 encode for Major Facilitator Superfamily proteins (MFS), and 21 genes encoded for Aquaporins (AQP). Furthermore, genes involved in Fe transport such as ZIP transporters (11 genes), Nicotianamine Synthase genes (9 genes), and Yellow Stripe1-Like and NRAMP transporters (3 genes each) were also annotated in the common DEG list. Regarding their expression, several genes showed conserved expression pattern under both drought and Fe deficiency. Indeed, several genes encoding for ABC transporters and HMA proteins were up-regulated under both stresses (Figure 3A). Notably, three genes encoding for YSL (TraesCS2B02G097400, TraesCS1B02G436500, and TraesCS1D02G414700) were up-regulated in both leaves and roots under both stress conditions (Figure 3A).

Several genes involved in iron uptake and transport in wheat were identified based on their orthologous relationship with their well characterized counterparts from Arabidopsis and rice [29]. We have examined their expression among shared DEG which led to the identification of 25 genes showing a conserved expression pattern under drought and Fe deficiency (Figure 3B). Indeed, two orthologs of *OsNRAMP2* (TraesCS4A02G050500 and TraesCS4D02G254100) were induced under both drought and Fe deficiency in roots (Figure 3B). It was demonstrated that *OsNRAMP2* encodes for a vacuolar transporter involved in Fe remobilization during germination and its knock-out resulted in delayed seed germination and chloroses in seedling leaves [30]. Similarly, the expression of TraesCS3B02G296100 genes was induced by both stresses, especially in roots. This gene was the ortholog of *AtBTSL1* from *Arabidopsis* and encodes for BRUTUS-LIKE E3 ligases which negatively regulate Fe uptake [31]. Interestingly, orthologs of *OsIRO3* TFs (TraesCS2A02G081300, TraesCS2B02G095900 and TraesCS2D02G079100) were also induced by drought and Fe deficiency (Figure 3B). *OsIRO3* belongs to BHLH TF family and negatively regulates Fe homeostasis in rice [32].

Conversely, several genes exhibited stress-specific expression patterns, with distinct induction under either drought or Fe deficiency condition, i.e., orthologs of *OsZIP5* (TraesCS1D02G293900 and TraesCS1A02G297500), *AtFER1* (TraesCS5B02G151000), and *AtHMA1* (TraesCS7B02G337700) were specifically up regulated by drought stress in roots while their expression was down-regulated under Fe deficiency (Figure 3B). These results showed that the expression Fe uptake genes were affected by drought and Fe deficiency in a tissue independent manner suggesting similar strategies for Fe uptake and transport under both stress condition.

### 2.4. Drought and Fe Deficiency Affect Phytohormone Signaling

**Figure 4:**
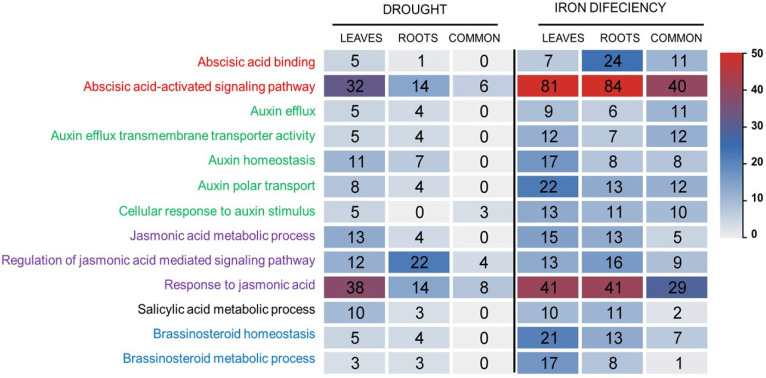
Numbers of differentially expressed hormone-related genes (HRGs) under drought and Fe deficiency based on GO term enrichment analysis.

GO term enrichment analysis showed that 190 and 512 genes involved in phytohormone pathways were modulated by drought and Fe deficiency, respectively (Figure 4).

Indeed, the highest number of HRGs were related to ABA. These genes were involved in ‘abscisic-acid-activated signaling pathway’ (GO:0009738) and ‘abscisic acid binding’ (GO:0010427) (Figure 4). Moreover, gene related to auxin were distributed into five GO terms such as ‘cellular response to auxin stimulus’ (GO:0071365), ‘auxin homeostasis’ GO:0010252), ‘auxin polar transport’ (GO:0009926), ‘auxin efflux’ (GO:0010315), and ‘auxin efflux transmembrane transporter activity’ (GO:0010329). In addition, 13 and 23 genes were related to salicylic acid metabolic processes (GO:0009696) under Fe deficiency and drought stress, respectively. Regarding genes involved in jasmonic acid signaling, those involved in the response to jasmonic acid were the most represented (Figure 4). Furthermore, 42 HRGs were common to drought and Fe deficiency from which 5 genes were induced under both stresses (TraesCS3D02G318600, TraesCS4A02G244500, TraesCS3B02G353000, TraesCS3B02G495700, and TraesCS7A02G001400). These genes were related to auxin, jasmonic acid, and salicylic acid GO terms (Supplementary Material Figure S2).

### 2.5. Transcription Factors Controlling Fe Uptake Under Drought and Fe Deficiency

**Figure 5:**
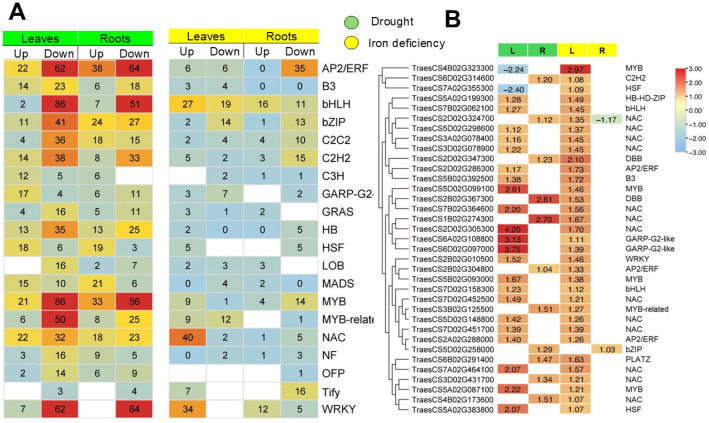
(**A**): Number and expression pattern of differentially expressed TFs in each gene family. (**B**): Heatmaps showing the expression pattern of conserved up-regulated TF encoding genes under drought and Fe deficiency. Empty boxes means that the gene was not differentially expressed in the corresponding tissue.

A total of 409 and 1416 DEGs encoding for TFs were identified under Fe deficiency and drought stress, respectively. Notably, 149 TF-encoding genes were differentially expressed in response to both stresses. Genes belonging to the AP2/ERF, MYB, bHLH, WRKY, NAC, and bZIP families were the most abundant (Figure 5A).

Among shared TF genes, 35 showed a highly conserved expression pattern under both stress conditions in the same tissue, from which one gene (TraesCS5D02G258000) belonging to the bZIP family was specifically induced under both stresses in roots. The remaining genes were mostly up-regulated in leaves. In addition, 22 genes were specifically induced in leaves, and they belong to NAC, B3, bHLH, MYB, and AP2/ERF families. Two GARP-G-Like encoding genes (TraesCS6A02G108800 and TraesCS6D02G097000) showed the highest expression (Log_2_FC > 3). The NAC TF family was highly represented among the up-regulated genes in leaves and roots under combined stress (Figure 5B) from which the gene TraesCS2D02G305300 showed the highest induction with a Log_2_FC > 4. Additionally, two members of the bHLH TF gene family (TraesCS7B02G062100 and TraesCS7D02G158300) showed specific expression in leaves under both stress conditions. indeed, the overall expression pattern of these TFs demonstrated tissue-specific response to drought and Fe deficiency which suggests synergistic regulation of iron homeostasis and drought stress.

### 2.6. Co-Expression Network Analysis Revealed Hub Gene for Fe Transport Under Drought

**Figure 6:**
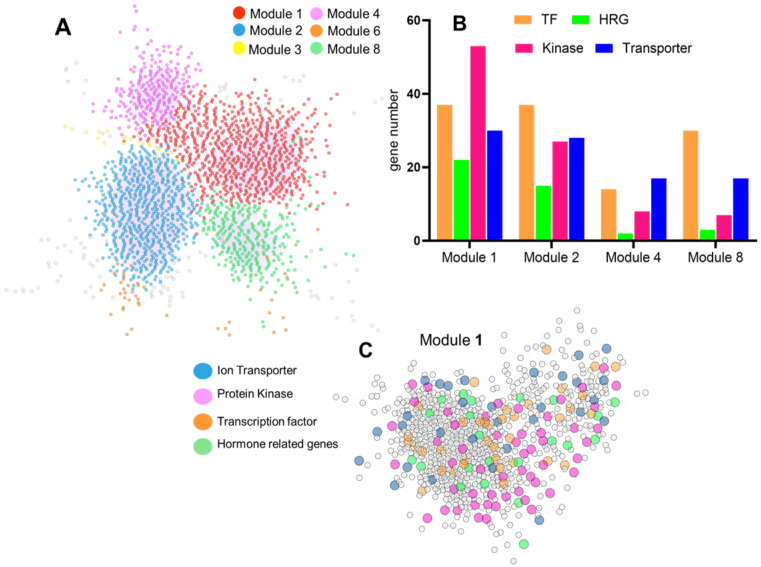
Co-expression network analysis of common DEGs. (**A**) Overview of the gene co-expression network constructed from common DEGs under drought and Fe deficiency; genes belonging to the same modules were highlighted by the same color. (**B**) Distribution of gene family across the identified modules. (**C**) Detailed overview of module 1; genes encoding for TFs, ion transporters, hormone-related genes (HRGs), and protein kinases were highlighted in different colors.

We carried out co-expression analysis using the normalized gene expression values of the common DEGs to investigate genes associated with the cross regulation of drought and Fe deficiency. Subsequently, to identify co-expression modules enriched with genes involved in similar signaling pathways, community clustering analysis using Glay was carried out and identified six major modules, each containing over 20 genes (Figure 6A). Four of these modules (modules 1, 2, 4, and 8) were significantly enriched with genes involved in transcription regulation, Fe homeostasis, the protein kinase pathway, and hormone signaling (Supplementary Material Figure S3). Notably, module 1 contained the highest number of Fe homeostasis genes, 37 TFs, and 30 ion transporters compared to the other modules (Figure 6B).

In order to investigate the biological processes within the identified co-expression modules, GO enrichment was carried out using the gene set of each module. Results (Supplementary Material Table S3) showed that module 1 was significantly enriched with GO terms directly related to Fe homeostasis, such as Fe ion binding (GO:0005506) and 2Fe-2S cluster binding (GO:0051537). Moreover, module 2 showed a broader functional scope, exhibiting significant enrichment for terms related to oxidative stress response, like hydrogen peroxide catabolic process (GO:0042744), peroxidase activity (GO:0004601), and response to oxidative stress (GO:0006979), alongside key Fe homeostasis terms, such as heme binding (GO:0020037) and ABC-transporter activity (GO:0140359). This module was also enriched for GO terms related to stress response and transcription regulation such as DNA-binding TF activity (GO:0003700) and transcription regulator activity (GO:0140110).

Collectively, the co-expression network analysis strongly supports the interaction between TFs and Fe homeostasis genes within these modules, suggesting their crucial role in maintaining Fe homeostasis in wheat under drought stress.

### 2.7. Validation of Candidate TFs by RT-qPCR

**Figure 7:**
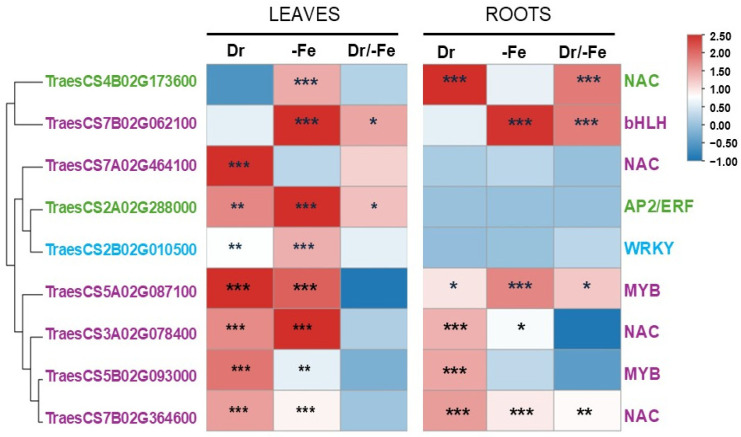
Expression analysis of candidate hub TFs. Heatmap showing the relative expression levels (Log_2_Fold Change, FC) of nine candidate hub TFs in leaves and roots under drought (Dr), Fe deficiency (-Fe), and combined stresses (Dr/-Fe). Expression levels were determined by qRT-PCR as described in the Methods section. Asterisks indicate statistical significance of differences compared to control plants (* *p* ≤ 0.05, ** *p* ≤ 0.01, *** *p* ≤ 0.001, Student’s *t*-test). Gene labels are color-coded according to their respective co-expression module shown in Figure 6.

To further confirm their role in stress response, a set of TF genes with the highest co-expression relationship with Fe homeostasis genes were selected to investigate their expression patterns in a local durum wheat genotype (cv Karim) under drought, Fe deficiency, and combined stresses in leaves and roots. Results of the RT-qPCR analysis confirmed the differential expression of the selected hub TFs, corroborating the RNA-seq data and suggesting conserved expression profiles across experiments (Figure 7).

In leaves, six genes were significantly induced by drought and Fe deficiency, including TFs belonging to the AP2/ERF, WRKY, NAC, and MYB families (TraesCS2A02G288000, TraesCS2B02G010500, TraesCS3A02G078400, TraesCS5A02G087100, TraesCS5B02G093000, and TraesCS7B02G364600) with expression ranging from 2- to 8-fold compared to the control (Figure 7). The expression of the AP2/ERF gene (TraesCS2A02G288000) was specifically induced by individual and combined stresses in leaves. Similarly, the WRKY TF (TraesCS2BG010500) was significantly up-regulated in leaves under individual stress in leaves while its expression was not affected in roots. Two genes (TraesCS4B02G173600 and TraesCS7B02G062100) showed Fe-dependent induction in leaves, but their expression was not modulated when drought was imposed indicating that drought could impact the regulation of Fe homeostasis pathways.

In roots, only six genes showed differential expressions from which three genes (TraesCS3A02G078400, TraesCS5A02G087100, and TraesCS7B02G364600) were induced by both stresses (Figure 7). Notably, the bHLH TF (TraesCS7B02G062100) was highly induced by both Fe deficiency and combined stresses in roots, mirroring its behavior in leaves (Figure 7).

Comparative expression analysis revealed distinct tissue-specific adaptation mechanisms to drought and Fe deficiency in wheat. For example, the NAC TF (TraesCS4B02G173600) was specifically induced by Fe deficiency in leaves and by drought and combined stresses in roots (Figure 7). Similarly, the MYB TF (TraesCS5B02G093000) was induced by both stresses in leaves, but this induction was restricted to drought in roots. Moreover, three genes (TraesCS7A02G464100, TraesCS2A02G288000, and TraesCS2B02G010500) were specifically up-regulated in leaves in response to individual or combines stresses (Figure 7). Considered together, the induction of these candidate TF genes under drought is heavily influenced by the plant’s Fe nutritional status, demonstrating a close regulatory crosstalk where low Fe availability modulates drought-induced responses.

## 3. Discussion

Drought and Fe deficiency are major abiotic stressors that significantly impact plant growth and yield. While the effects of these stresses on plants have been extensively studied individually, their combined impact, as well as the molecular crosstalk between their signaling pathways, remains poorly understood. Unravelling this intricate interplay is crucial for developing effective strategies to mitigate the negative impacts of these stresses on crop production. Here, we carried out a comparative transcriptomic analysis of wheat responses to drought and Fe deficiency to elucidate the regulatory components involved in Fe homeostasis during drought stress.

Our initial transcriptome data mining identified 2479 genes that were differentially expressed under both drought and Fe deficiency, strongly suggesting common signaling mechanisms under these two stress conditions. Leveraging this overlap, we employed a methodology successfully used in previous studies to identify complex regulatory networks, such as using RNA-seq data mining in combination with co-expression network analysis to identify multiple abiotic stress candidate genes and master regulators in wheat and rice [33].

Fe homeostasis in plants is maintained by several processes involving genes related to high-affinity uptake, transport and distribution, ligand synthesis, and regulatory factors [34]. A closer examination of the expression patterns of genes encoding transporter families revealed that ABC transporter, Aquaporin, and proteins such as Heavy-Metal-Associated (HMA) domain proteins were induced by both drought and Fe deficiency (Figure 3). Notably, ABC transporters are ubiquitous in living organisms, mediating the transport of various essential compounds in plants, including ABA, secondary metabolites, ROS, and heavy metal ions [35].

Plant hormones play a crucial role in stress sensing in plants. Our GO terms enrichment analysis revealed that several HRGs were differentially expressed, especially those involved in ABA and jasmonic acid pathways (Figure 4). The role of ABA is critical in this crosstalk: in *Arabidopsis*, ABA alleviates Fe deficiency symptoms by promoting Fe uptake and transport [22] and several ABA-responsive genes are induced by Fe deficiency via the IDEF1 transcription factor in rice [36]. This connection is further supported by evidence suggesting cross-connections between Fe deficiency and ABA signaling under osmotic stresses, involving key regulators like FIT, IRT1, and ABA response protein ABI5 [37]. These findings strongly suggest that HRGs and transcription regulators were involved in the Fe uptake network under osmotic stresses and involved an ABA-dependent response.

Consistent with this regulatory focus, we investigated the expression of genes known for their roles in Fe uptake and transport (Figure 3). We found that orthologs of *OsIRO3* and *OsNRAMP2* were induced by both stress conditions. Notably, *OsIRO3* (*OsbHLH63*) is a known negative regulator of Fe homeostasis that represses *OsIRO2* [32]. Moreover, orthologs of Yellow Stripe-Like (YSL) proteins (*OsYSL15*, *OsYSL12*, *AtYSL4)* were modulated by both drought and Fe deficiency. Indeed, YSL proteins are crucial for Fe uptake and are typically strongly induced by Fe deficiency [38]. It was reported that *osysl15* mutants exhibited typical Fe deficiency symptoms and contained lower Fe content compared to overexpressing lines [39]. Similar observations were reported for *AtYSL4* cooperating with *AtYSL6* in Fe mobilization from the chloroplast [38].

Stress-responsive genes, particularly those encoding TFs and hormone response, are key regulators of plant responses to single or multiple abiotic stresses. The stress-specific expression patterns of these genes serves as signatures of the prevailing stress conditions. Our analysis showed that several TF-encoding genes were modulated by both drought and Fe deficiency, including members of the NAC, bHLH, AP2/ERF, and MYB families. Furthermore, co-expression network analysis identified hub TFs that exhibited strong correlation with genes encoding Fe transporters, HRGs, and protein kinases. These findings strongly suggest an intricate interconnection between drought and Fe deficiency response pathways. Indeed, the *OsIRO2*, a bHLH TF, regulates the expression of several Fe transport-related genes, including *OsNAS1*, *OsNAS2*, *OsYSL15*, and *OsNAAT1* [11], and its expression is regulated by another bHLH TF, *OsIRO3* [32]. Consistent with this established role, we found that orthologs of *OsIRO3* were induced by both drought and Fe deficiency (Figure 3). These results suggest that Fe deficiency bHLH landmark genes could be crucially involved in regulating Fe homeostasis under drought stress. Moreover, the Fe deficiency-responsive bHLH protein, also known as FIT (Fer-Like Fe Deficiency-Induced TF), further underscores this pathway, regulating the expression of key Fe transporting genes such as *AHA2*, *FRO2*, and *IRT1* [17].

In addition to bHLH TFs, members of the NAC, MYB, and WRKY TF families are widely recognized for their involvement in abiotic stress responses [40,41,42]. Consistent with this, our study identified NAC and MYB genes that were induced by both drought and Fe deficiency. The expression of these TF genes was further validated by RT-qPCR analysis in durum wheat (Figure 7) and, critically, these genes belong to a co-expression module enriched with genes involved in Fe transport, suggesting a direct regulatory link. This link mirrors findings such as the *Arabidopsis* MYB88 genes, which is reported to regulate Fe deficiency by binding a specific motif in the IRT1 promoter [16]. Furthermore, the NAC TFs from durum wheat (*TtNAC2A* and *TtNTL3A*) have been shown to enhance drought tolerance through an ABA dependent pathway and enhanced the expression of drought tolerance genes in transgenic *Arabidopsis* [43,44].

Our expression analysis demonstrated that the expression of several candidate TFs were organ-specific, suggesting specific adaptation mechanisms in wheat responding to drought and Fe deficiency. The NAC TF (TraesCS4B02G173600) was specifically induced by Fe deficiency in the leaves, the primary sites of photosynthesis, suggesting its role in systemic Fe signaling or nutrient recycling. Conversely, its induction by drought and combined stresses, but not by Fe deficiency, in the roots points its primary function toward root-specific resilience, such as maintaining water uptake capacity or mitigating dehydration damage [40,41,42]. Similarly, the MYB TF (TraesCS5B02G093000) was induced by both Fe deficiency and drought in leaves, supporting the role of MYB transcription factors in broad shoot protection by regulating cuticular wax for water conservation [45,46]. Crucially, its restriction to drought induction in the roots suggests its exclusion from the core Fe acquisition machinery, focusing its role strictly on root-level drought adaptation. Furthermore, the significant up-regulation of three genes (TraesCS7A02G464100, TraesCS2A02G288000, and TraesCS2B02G010500) in the leaves under stress conditions highlights the plant’s strategy to protect the shoot integrity by the rapid induction of specialized mechanisms related to photoprotection, ROS detoxification, or water conservation.

Finally, the regulatory overlap between stresses is further highlighted by the AP2/ERF family. Besides their known role in drought, AP2/ERF genes are implicated in Fe metabolism. For example, the AP2/ERF gene (*AtERF4*) negatively regulates the Fe deficiency response in *Arabidopsis* by repressing *AtIRT1* and *AtHA2* [47]. Consistent with this, our study identified a hub AP2/ERF gene in co-expression module 8 that was induced by both drought and Fe deficiency (Figure 7). The strong induced expression of several hub TFs, including AP2/ERF, NAC, bHLH, and WRKY family members, especially in leaves, strongly suggests their critical regulatory role in maintaining wheat Fe homeostasis under drought stress.

In summary, we performed a comprehensive regulatory network analysis to identify regulatory genes belonging to the co-expression network underlying the coordinated wheat response to drought and Fe deficiency. Our study successfully generated valuable genetic resources for improving wheat resilience to both stresses. However, future work is essential to further understand their role in stress response, and should include the functional validation of candidate TFs through overexpression studies and the analysis of the physiological and molecular response of transgenic lines to both individual and combined stress conditions.

## 4. Materials and Methods

### 4.1. Plant Material Stress Treatment

Durum wheat (*Triticum turgidum* L. ssp. Durum, cv. Karim) seeds were germinated on wet filter paper. Seven-day-old seedlings were then transferred to full strength Hoagland hydroponic solution under standard greenhouse conditions for seven days. Drought stress was imposed by transferring plants to nutrient solution supplemented with 10% (*w*/*v*) PEG 6000 for seven days while Fe deficiency by transferring plants to nutrient solution containing 10 μM Fe(III)-EDTA for 3 weeks. For combined stress, half of the plants cultivated in Fe-limited conditions (two weeks) were subjected to drought as described above for 7 days. The nutrient solutions for both control and stress treatments were refreshed weekly with continuous air pumped into the hydroponics solution. Leaves were collected, frozen in liquid nitrogen, and stored at −80 °C until RNA extraction. Three biological replicates were collected for each sample.

### 4.2. Expression Analysis of Candidate Genes by qRT-PCR

Total RNA was extracted using the EZ-10 Spin Column Plant RNA Miniprep Kit (BIOBASIC, New York, NY, USA) following the manufacturer’s instructions. First-strand cDNA was synthesized from two micrograms of total RNA using the 5X All-In-One RT PCR Master Mix (BIOBASIC). qRT-PCR assays were carried out on the CFX96 Touch Real-Time PCR Detection System (Bio-rad, Hercules, CA, USA) using the GoTaq^®^ qPCR Master Mix (Promega, Madison, WI, USA, Catalog number: A6001) following the manufacturer’s instructions. Relative expression was normalized against the reference gene encoding for actin and calculated using the 2^−∆∆Ct^ method. Primers for each gene (Supplementary Table S4) were designed using Primer 3 software. Three biological replicates were carried, and each replicate sample consisted of pooled tissues from three plants cultivated under the same conditions.

### 4.3. Identification of Differentially Expressed Genes (DEGs)

RNA-seq from bread wheat var. Chinese Spring subjected to drought stress for 7 days (NCBI accession number: SRP098756) and Bobwhite S26 genotypes subjected to Fe deficiency (10 µM Fe) for 90 days (NCBI accession number: SRP293922) were used for differentially expressed genes retrieval. Both transcriptomic data were generated using Illumina sequencing. The data was processed and mapped to the WGSC wheat genome assembly RefSeq v1.1 as previously described [48]. Indeed, Gene expression levels (TPM, transcript per million) were retrieved from PlantExp (https://biotec.njau.edu.cn/plantExp) database (Accessed 1 April 2023; Supplementary Table S1). Genes with expression value lower than 0.5 were removed and the DEGseq2 package (version 1.50.1) [49] was used for the identification of significant DEGs. Only genes with at least two-fold change in expression (False discovery rate *FDR* < 0.05) were considered as significant. The FDR was calculated using the Benjamini and Hochberg [50] method. Moreover, Venn diagrams were used to identify common and specific DEGs using Venny server (https://bioinfogp.cnb.csic.es/tools/venny/index.html, Version 2.1).

### 4.4. Functional Enrichment Analyses

The biological function of the DEGs was analyzed using the ShinyGO (http://bioinformatics.sdstate.edu/go/, Version 0.85.1) database with the following parameters: *p* value < 0.05, FDR correction by the Hochberg method.

### 4.5. Co-Expression Network Analysis

Hierarchical clustering analysis of expression data was carried out with TBtool software (version 2.2010) [51]. The gene co-expression network was constructed from the gene expression matrix using the “ExpressionCorrelation” plunging and the ClusterMaker2 plunging [52] was used for the identification of co-expression modules with “community clustering” (GLay). Network manipulation and visualization was carried out in Cystoscape 3.10 software [53].

### 4.6. Data Analysis

Data were presented as means values ± SD of three biological replicates. GraphPad Prism 10 software (GraphPad Software, San Diego, CA, USA) was used for data visualization. Statistical analyses were carried out using SAS JMP Pro 17 software.

## 5. Conclusions

Our RNA-seq data analysis successfully identified differentially expressed genes under both drought and Fe deficiency across leaves and roots. Comparative analysis revealed a significant overlap in both stress signaling pathways including hormone signaling, Fe transport, and transcriptional regulation. Notably, TF genes showed conserved up-regulation in leaves compared to roots. Furthermore, the gene co-expression network elucidated the gene relationships involved in transcriptional regulation, iron homeostasis, and stress response. Several transcription factors, especially from the NAC, AP2/ERF, and bHLH families, actively reacted to drought and iron deficiency and might play a crucial role in linking hormone signaling and Fe transport under drought stress. This work provides an important genetic resource for further functional investigation, offering promising targets to enhance Fe uptake and achieve biofortification in wheat under drought stress.

## Data Availability

The original contributions presented in this study are included in the article/Supplementary Material. Further inquiries can be directed to the corresponding authors.

## Supplementary Materials

The following supporting information can be downloaded at: https://www.mdpi.com/article/10.3390/ijms262210955/s1.
