# Supplementary material for: Transcriptomics Data Mining to Identify Novel Regulatory Genes of Iron Uptake in Drought-Stressed Wheat
Source: Int J Mol Sci. 2025 Nov 12;26(22):10955. doi: 10.3390/ijms262210955 (PMC12652351; doi:10.3390/ijms262210955)
Supplement: Supplementary file 1 [file ijms-26-10955-s001.zip › Supplementary Figures saidi el 2025.pdf]

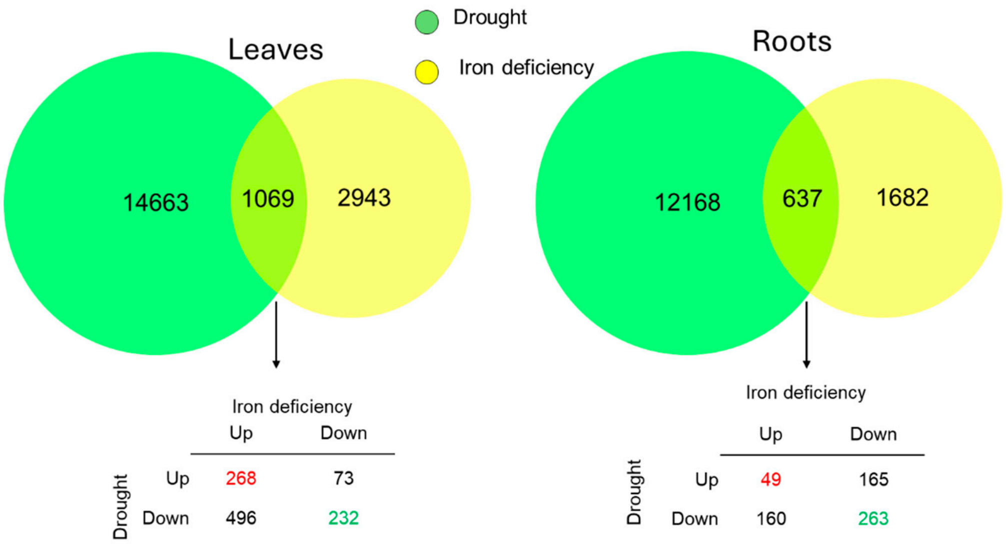

Supplementary Figure S1. Two-way Venn diagrams showing conserved DEGs in leaves and roots under drought and iron deficiency.

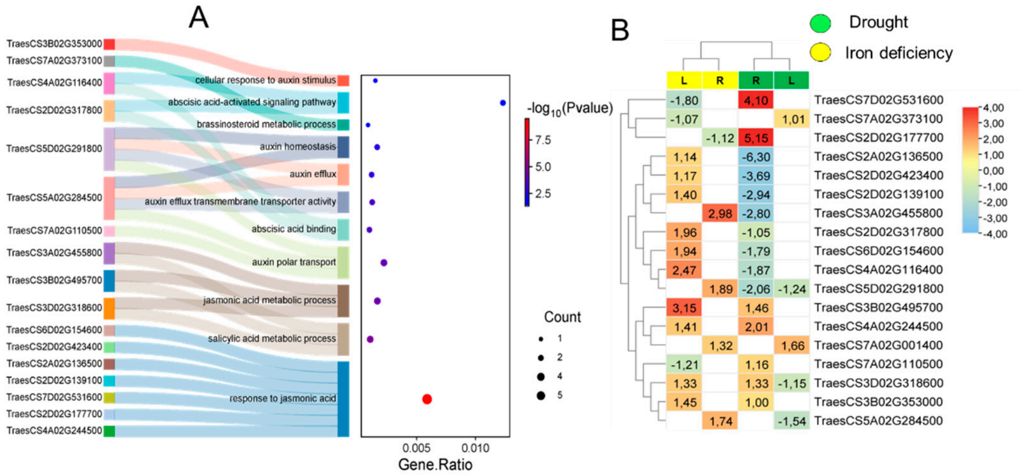

Supplementary Figure S2. **A**: Upregulated HRGs under drought and Fe deficiency and their enriched GO terms. **B**: Expression pattern of common up-regulated HRGs.

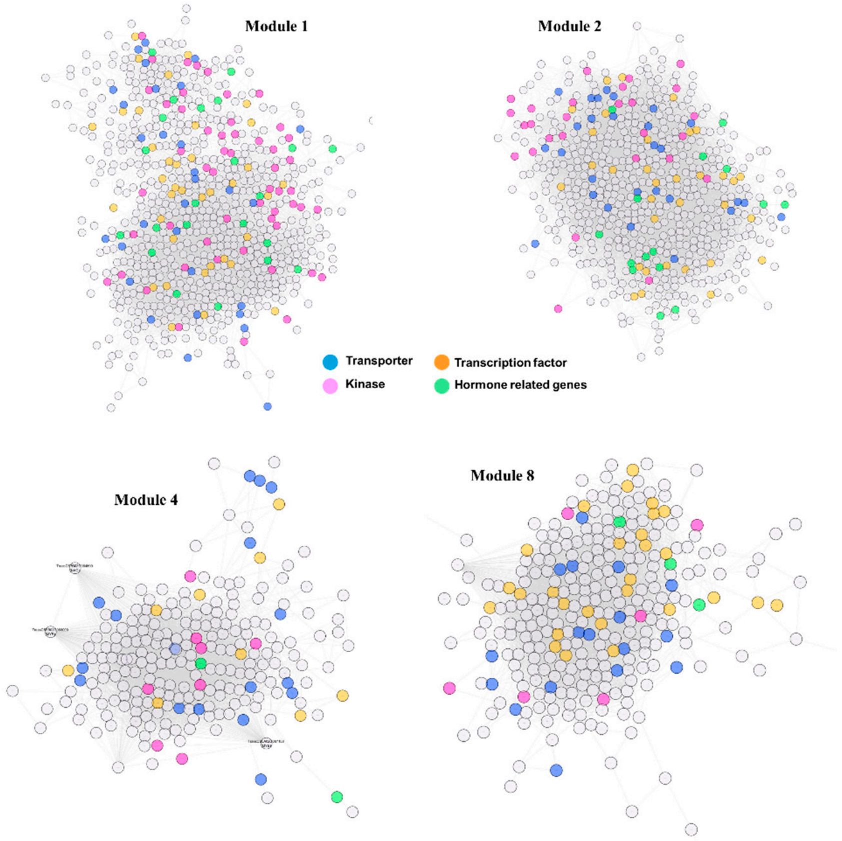

Supplementary Figure S3. Co-expression modules enriched with genes encoding for transcription factors, Protein kinases, transporters and Hormone-related genes.
