# Supplementary material for: Transcriptomics Data Mining to Identify Novel Regulatory Genes of Iron Uptake in Drought-Stressed Wheat
Source: Int J Mol Sci. 2025 Nov 12;26(22):10955. doi: 10.3390/ijms262210955 (PMC12652351; doi:10.3390/ijms262210955)
Supplement: Supplementary file 1 [file ijms-26-10955-s001.zip › Supplementary Table S4 primers sequences.pdf]

**Supplementary Table S4**: primers used for RT-qPCR

| GeneID | Forward | Reverse |
|---|---|---|
| **TraesCS2A02G288000** | ATCAGTGGATCGAAGGTGCT | TGTCATATTAGCCACATTATTGCAT |
| **TraesCS2B02G010500** | GCACGCTCGCAGAGATTAT | TCGCATGAGTAACCTGCATC |
| **TraesCS3A02G078400** | GCGATTTGACTATTGCCACAC | ATATCCCCCACGATCCATTG |
| **TraesCS4B02G173600** | GGCGAACTGGTGATTTGAAG | ACACACACCTGCAGTACAATCC |
| **TraesCS5A02G087100** | TCCTCATACGGACTTGTTCTCC | GCGCTTGTACATTTGCATCC |
| **TraesCS5B02G093000** | TATATGTTGGAAAAGGGTACGTG | GACGCATCTAATTCAACACCAC |
| **TraesCS7A02G464100** | AGGAAAAGGAGGGTCAGAGG | CGTCTTATCCATAGTCGCACAC |
| **TraesCS7B02G062100** | CTAAAATCCGAGGCTGAAGG | ATACACACCAACCGATGTGC |
| **TraesCS7B02G364600** | GCTATTGCATGCAGTTGTGG | TCTCCTCTGACCCTCCTTTTC |
| **Actin** | ACCTTCAGTTGCCCAGCAAT | CAGAGTCGAGCACAATACCAGTTG |
